# Follicle Size on Day of Trigger Most Likely to Yield a Mature Oocyte

**DOI:** 10.3389/fendo.2018.00193

**Published:** 2018-04-25

**Authors:** Ali Abbara, Lan N. Vuong, Vu N. A. Ho, Sophie A. Clarke, Lisa Jeffers, Alexander N. Comninos, Rehan Salim, Tuong M. Ho, Tom W. Kelsey, Geoffrey H. Trew, Peter Humaidan, Waljit S. Dhillo

**Affiliations:** ^1^Imperial College London, Hammersmith Hospital, London, United Kingdom; ^2^University of Medicine and Pharmacy at Ho Chi Minh City, Ho Chi Minh City, Vietnam; ^3^IVFMD, My Duc Hospital, Ho Chi Minh City, Vietnam; ^4^IVF Unit, Hammersmith Hospital, London, United Kingdom; ^5^School of Computer Science, University of St Andrews, St Andrews, United Kingdom; ^6^The Fertility Clinic, Skive Regional Hospital, Faculty of Health, Aarhus University, Aarhus, Denmark

**Keywords:** follicle size, trigger, mature oocyte, IVF treatment, kisspeptin

## Abstract

**Objective:**

To identify follicle sizes on the day of trigger most likely to yield a mature oocyte following hCG, GnRH agonist (GnRHa), or kisspeptin during IVF treatment.

**Design:**

Retrospective analysis to determine the size of follicles on day of trigger contributing most to the number of mature oocytes retrieved using generalized linear regression and random forest models applied to data from IVF cycles (2014–2017) in which either hCG, GnRHa, or kisspeptin trigger was used.

**Setting:**

HCG and GnRHa data were collected at My Duc Hospital, Ho Chi Minh City, Vietnam, and kisspeptin data were collected at Hammersmith Hospital, London, UK.

**Patients:**

Four hundred and forty nine women aged 18–38 years with antral follicle counts 4–87 were triggered with hCG (*n* = 161), GnRHa (*n* = 165), or kisspeptin (*n* = 173).

**Main outcome measure:**

Follicle sizes on the day of trigger most likely to yield a mature oocyte.

**Results:**

Follicles 12–19 mm on the day of trigger contributed the most to the number of oocytes and mature oocytes retrieved. Comparing the tertile of patients with the highest proportion of follicles on the day of trigger 12–19 mm, with the tertile of patients with the lowest proportion within this size range, revealed increases of 4.7 mature oocytes for hCG (*P* < 0.0001) and 4.9 mature oocytes for GnRHa triggering (*P* < 0.01). Using simulated follicle size profiles of patients with 20 follicles on the day of trigger, our model predicts that the number of oocytes retrieved would increase from a mean 9.8 (95% prediction limit 9.3–10.3) to 14.8 (95% prediction limit 13.3–16.3) oocytes due to the difference in follicle size profile alone.

**Conclusion:**

Follicles 12–19 mm on the morning of trigger administration were most likely to yield a mature oocyte following hCG, GnRHa, or kisspeptin.

## Introduction

IVF treatment involves the administration of supra-physiological doses of follicle-stimulating hormone (FSH) to induce the growth of multiple ovarian follicles. Once ovarian follicles grow to an appropriate size, a trigger is administered to mature the oocytes in preparation for oocyte retrieval. It is widely accepted that ovarian follicles that are “too small” are less likely to respond suitably to trigger administration and yield a mature oocyte (1). Furthermore, once ovarian follicles grow too large, follicles may contain oocytes that are “post-mature” and also not competent for fertilization (2). Most IVF centers will therefore, monitor follicular size and administer the trigger of oocyte maturation once follicles are deemed to have grown to an appropriate size.

Relevant data exist as to the appropriate size of follicles on the day of oocyte retrieval that are most likely to yield an oocyte in both human and animals models (1). Overall, follicles of 16–22 mm on the day of oocyte retrieval are more likely to contain mature oocytes than smaller follicles, while larger follicles are more likely to contain post-mature oocytes (1). However, limited data exist to establish which follicle size on the day of trigger is most likely to yield a mature oocyte.

Data on follicle size on day of trigger with greatest propensity to yield oocytes are suggested by Hu and colleagues (3). They categorized Chinese women co-treated with a GnRH antagonist cycles by the proportion of follicles ≥10 mm which were also ≥17 mm on the day of trigger; as low (30% ≥17 mm), middle (30–60% ≥17 mm), or high proportion (>60% ≥17 mm) (3). The investigators determined that the number of oocytes retrieved was greatest in those with a low proportion of follicles ≥17 mm (9.2 low vs 7.6 middle and 7.2 high) (3).

Importantly, knowledge of the size of follicles on day of trigger from which one could reasonably expect to retrieve a mature oocyte could enable the accurate determination of trigger efficacy. In 2007, Shapiro et al. compared the efficacy of hCG and GnRH agonist (GnRHa) triggering (4), observing that GnRHa use resulted in significantly more oocytes retrieved (28.8) when compared with hCG (21.6) (4). However, patients receiving GnRHa had a greater number of follicles on the day of trigger (GnRHa 34.2 follicles; hCG 21.7 follicles) making it difficult to accurately compare trigger efficacy between the two groups (4). Thus, in their later work, Shapiro introduced the concept of an “oocyte yield,” whereby the number of oocytes collected is corrected for the number of follicles on the day of trigger (5). They reported mature oocyte yields (mature oocytes from follicles of ≥10 mm) of 63% after GnRHa (5). Other authors have reported both number of follicles ≥14 mm and the number of follicles ≥10 mm to allow the reader to account for different estimations of oocyte yield (6).

Kisspeptin is an endogenous neuropeptide that plays a key role in regulating the hypothalamo–pituitary–gonadal axis (7). Collectively, data from both animal models and humans have demonstrated that exogenous kisspeptin administration stimulates endogenous GnRH release from the hypothalamus (7). Recently, kisspeptin has been used to induce oocyte maturation during IVF cycles with low rates of OHSS even in high risk women (8). Studies evaluating kisspeptin as a trigger of oocyte maturation used a denominator of follicles ≥14 mm on day of trigger to compare trigger efficacy following different doses and demonstrated a reasonable dose-response (9). Importantly, none of the denominators used to date are evidence-based, nor do they have an upper limit for follicle size to account for follicles containing post-mature oocytes.

Therefore, we sought to determine the size of follicles on day of trigger that would be most likely to yield a mature oocyte. To identify the follicle sizes which were most likely to yield a mature oocyte, we analyzed follicle size data from 499 IVF cycles triggered with either hCG, GnRHa, or kisspeptin.

## Materials and Methods

### Study Participants

Women were aged 18–38 years with a body mass index (BMI) 18–29 kg/m^2^ and had antral follicle counts 4–87. GnRHa data were from a randomized controlled trial and hCG data from a case-series conducted at My Duc Hospital, Ho Chi Minh City, Vietnam (10). Kisspeptin data were obtained from patients undergoing clinical trials at Hammersmith Hospital, London (9, 11, 12).

#### GnRHa and hCG Triggers

Data for GnRHa were obtained from a randomized controlled trial of triptorelin dose 0.2–0.4 mg conducted at My Duc Hospital, Ho Chi Minh City, Vietnam. Data for hCG trigger were from a case-series also carried out at My Duc Hospital, Ho Chi Minh City, Vietnam. Inclusion criteria: age 18–38 years, BMI < 28 kg/m^2^, normal ovarian reserve: AMH > 1.25 ng/ml (8.93 pmol/l) or AFC ≥6 (13). Exclusion criteria: polycystic ovary syndrome, chronic medical condition, participating in another clinical trial or use of LH/FSH preparations prior to the study. Patients did not receive hCG if there were more than 20 follicles of ≥14 mm on the day of trigger.

#### Kisspeptin Trigger

Data were obtained from patients undergoing clinical trials at Hammersmith Hospital, London. Inclusion criteria: aged 18–34 years, BMI 18–29 kg/m^2^, early follicular FSH ≤12 IU/l, serum AMH ≥10 pmol/l (≥1.4 ng/ml), both ovaries intact. Exclusion criteria: moderate/severe endometriosis, poor ovarian response in a former IVF cycle [previous poor response (≤3 oocytes retrieved on a previous IVF cycle), or ≥2 previous IVF treatment cycles].

### Study Approvals

Data included in this manuscript were obtained from studies carried out in accordance with the recommendations of the local ethical boards listed below. All subjects gave written informed consent in accordance with the Declaration of Helsinki and Good Clinical Practice.

Data from GnRHa triggered IVF cycles were obtained from a single-center randomized controlled trial conducted at My Duc Hospital, Ho Chi Minh City, Vietnam (10). The Institutional Review Board (IRB) reference number was NCKH/CGRH_01_2014 and ClinicalTrials.gov registration was NCT02208986. For the hCG case-series, the IRB reference number was NCKH/CGRH_09_2017, ethical approval reference number: 10/17/DD-BVMD and ClinicalTrials.gov Identifier: NCT03174691. For the kisspeptin trial ethical approval was granted by the Hammersmith and Queen Charlotte’s Research Ethics Committee, London, UK (reference: 10/H0707/2), undertaken at the IVF Unit at Hammersmith Hospital under a license from the UK Human Fertilization and Embryology Authority (9, 11, 12) and registered on the National Institutes of Health Clinical Trials database (NCT01667406).

### IVF Protocol

Full details of the IVF protocols used for the GnRHa study (10) and the kisspeptin study (9, 11, 12) have previously been reported. In short, all IVF cycles were conducted using GnRH antagonist co-treatment and the trigger was administered once two to three follicles reached 17–18 mm in diameter. All follicles that were visible on ultrasound and ≥8 mm in diameter were aspirated at oocyte retrieval. Flushing was not conducted in GnRHa or hCG-triggered cycles. Flushing was occasionally conducted in kisspeptin-triggered cycles, although the literature suggests that this is unlikely to have impacted the number of oocytes retrieved (14, 15).

Patients triggered with GnRHa were stimulated, using a depot injection of 100–150 IU of corifollitropin alfa (Elonva; Merck Sharp & Dohme, UK) from cycle day 2, followed by co-treatment with ganirelix (Merck Sharp & Dohme B.V., Germany) (starting on day 5 after stimulation) and follitropin-β. The corifollitropin alfa dose used for stimulation was either 100 or 150 µg, depending on body weight, and the corresponding follitropin-β dose was 150 or 200 IU/day, starting from day 8 of simulation until the day of triggering. Patients triggered with hCG were stimulated with follitropin-β daily (dose of 150–300 IU based on AMH level, AFC, age, history of previous response). The cycles were part of research conducted to study the endocrine profiles following triggering. The trigger (either recombinant hCG 250 µg equivalent to 6,500 IU, or GnRHa triptorelin 0.2–0.4 mg) was administered as soon as two follicles reached a size of ≥17 mm. Fresh embryo transfer was not carried out in GnRHa- or hCG-triggered cycles.

For kisspeptin-triggered cycles, recombinant FSH (112.5–150 IU Gonal F, Merck Serono, Geneva, Switzerland) was used to induce follicular growth and the GnRH antagonist cetrorelix (0.25 mg, Cetrotide, Merck Serono, UK) was administered from day 5 or 6. The trigger kisspeptin-54 (6.4–12.8 nmol/kg as a single subcutaneous bolus or 19.2 nmol/kg as a split bolus over 10 h, Bachem Holding AG, Bubendorf, Switzerland) was administered once three follicles reached ≥18 mm in diameter (9, 11, 12).

### Sizing Follicles on Day of Trigger

All patients included had a final ultrasound scan to assess follicle sizes on the morning of trigger. Sizing follicles was carried out during ultrasound assessment. For hCG and GnRHa triggered cycles, follicle size was assessed by transvaginal ultrasound (7.5 MHz probe) conducted by two dedicated ultrasonographers who have more than 10 years of follicle tracking, with a high degree of inter-observer correlation. For kisspeptin-triggered cycles, transvaginal ultrasonographic measurement (Toshiba Xario Prime, Crawley, UK) of follicle size was conducted by up to nine experienced IVF physicians/ultrasonographers at Hammersmith IVF unit over the 3-year study period.

### Statistical Analysis

Analysis was performed in three stages. Combinations of follicle sizes were calculated, e.g., number of follicles 8, 8–9, 8–10, 8–11, etc. This was repeated from a baseline of 9 mm, then 10 mm, and so on such that every possible category of follicular size was derived. Initially, standard linear regression of number of follicles of different size categories on day of trigger and outcomes (number of mature oocytes collected) was performed. This involved fitting linear models that identify the coefficient of determination (*r*^2^) between the number of follicles within a certain size range and the number of mature oocytes retrieved. The coefficient of determination describes the variability in the number of mature oocytes retrieved by the number of follicles within each follicle size range around a linear relationship. This provided initial confirmation that the number of follicles of different size ranges was associated with the number of mature oocytes retrieved following each trigger.

However, a simple linear model compares the number of follicles in each size category with the number of mature oocytes retrieved in isolation. Furthermore, simple linear models are susceptible to “autocorrelation,” whereby the number of follicles in one size category may also be included in other size categories. Hence, the more robust approach of generalized linear regression was used (16), allowing identification of the follicle size on the day of trigger with the greatest contribution to the number of mature oocytes retrieved, when compared with all other follicle size categories.

We used a third approach termed “random forest model,” which is a type of “ensemble modeling” utilizing modern machine learning technology (17). It is based on the formation of numerous decision trees to predict an outcome variable (in this case number of mature oocytes). Random forest models can be advantageous over generalized linear regression models if the number of outcome variables is comparatively low compared with the number of predictor variables. Random forest models also make no assumptions regarding linearity or parametric distributions in the data analyzed (and thus are less reliant on appropriate data transformation) and promote model variance by repeated sampling of the data. Hence, this method will more accurately return the follicle sizes with the greatest overall contribution to the number of mature oocytes retrieved. In our analysis 5,000 regression trees were produced, each derived using boot-strapped data (i.e., datasets of the same size as the entire data, but produced by random sampling with replacement), and the associations of number of follicles of different sizes with the number of mature oocytes were averaged across these models. More than one statistical approach was used to increase confidence that the data allowed accurate determination of optimal follicle size on day of trigger.

Statistical analysis was performed using R version 3.3.1; random forest models were derived using the randomForest pckage, and validated using the cforest package, which is designed to identify and correct for potential autocorrelations in the data used.

To quantify the follicle size profile benefit on the number of oocytes and mature oocytes retrieved for the subjects within our data set, we compared patients with a lesser proportion (<70%) of follicles within the follicle size range determined above to patients with all (100%) of their follicles within that follicle size range. To estimate the potential impact of follicle size profile, we simulated patients with varying follicle profiles and calculated the differences in the number of mature oocytes collected.

## Results

Baseline characteristics are presented in Table 1, which have in part previously been reported (9–12).

**Table 1:** Baseline characteristics.

	Trigger			
	hCG (*N* = 161)	GnRHa (*N* = 165)	Kisspeptin (*N* = 173)	*P*-value
Age (years)	32.3 ± 3.2	27.0 ± 4.3	30.5 ± 2.8	*P* < 0.001
Ethnicity	Southeast Asian 100%	Southeast Asian 100%	Caucasian 62.6% South Asian 28.2% Afro-Caribbean 4.6% Other 4.6%	*P* < 0.001
Weight (kg)	50.3 ± 5.7	51.3 ± 7.4	64.4 ± 9.4	*P* < 0.001
Body mass index (BMI) (kg/m^2^)	20.5 ± 2.1	20.8 ± 2.7	24.2 ± 3.2	*P* < 0.001
Serum AMH (ng/ml)	4.2 (26, 5.9)	6.4 (4.6, 9.4)	6.1 (3.5, 9.5)	*P* < 0.001
Antral follicle count	7 (5, 10)	17 (13, 24)	31 (25, 44)	*P* < 0.001
Number of follicles on day of trigger	14 (11, 16)	17 (13, 24)	27 (21, 39)	*P* < 0.001
Cumulative dose of recombinant follicle-stimulating hormone (IU)^a^	2,400 (2,025, 2,700)	900 (700, 1,300)	1,750 (1,388, 4,225)	*P* < 0.001

*Parametric variables are presented as mean ± SD while non-parametric are presented as median (interquartile range)*.

*^a^Patients triggered with hCG were stimulated using daily follitropin-β (150–300 IU daily), whereas patients triggered with GnRH agonist (GnRHa) received a depot injection of corifollitropin alfa (Elonva) followed by daily follitropin-β. Patients triggered with kisspeptin received a starting daily injection of corifollitropin alfa (Gonal F) of 112.5–150 IU. Patients who received GnRHa or kisspeptin had higher AMH levels and more follicles on the day of trigger. Patients who received hCG or GnRHa were from Southeast Asia with lower weight/BMI than patients who received kisspeptin in UK. Groups with continuous variables were compared by Kruskal–Wallis test and categorical variables by χ^2^ test*.

### Follicle Size on Day of Trigger With Greatest Contribution to the Number of Oocytes Retrieved—Simple Linear Regression Analysis

First-pass analysis using simple linear regression of the number of oocytes retrieved vs the number of follicles on day of trigger in each category of follicle size determined that the highest coefficient of determination to be at follicle size category 12–17 mm for hCG *r*^2^ 0.433 (*P* < 0.0001); 10–14 mm for GnRHa *r*^2^ 0.512 (*P* < 0.0001); and 7–18 mm for kisspeptin *r*^2^ 0.246 (*P* < 0.0001).

Simple linear regression of number of mature oocytes vs number of follicles in each category of follicle size determined that the highest coefficient of determination to be at follicle size category 12–17 mm for hCG *r*^2^ 0.374 (*P* < 0.0001); 10–15 mm for GnRHa *r*^2^ 0.573 (*P* < 0.0001); 7–20 mm for kisspeptin *r*^2^ 0.179 (*P* < 0.0001).

A further subset of patients triggered with kisspeptin had their final follicle monitoring scan on the day prior to the day of trigger administration (*n* = 62). For these patients, the highest coefficient of determination for the number of oocytes retrieved was for follicle size category 9–14 mm *r*^2^ 0.478 (*P* < 0.0001), and for the number of mature oocytes also for follicle size category 9–14 mm *r*^2^ 0.444 (*P* < 0.0001).

### Follicle Size on Day of Trigger With Greatest Contribution to the Number of Oocytes Retrieved—Generalized Linear Regression Model and Random Forest Model

A generalized linear regression model was used to determine the sizes of follicles on the day of trigger contributing most to the number of oocytes and mature oocytes retrieved (see Table 2). For both hCG and GnRHa, follicle sizes of 12–19 mm had the greatest contributions to the number of oocytes and mature oocytes retrieved (see Table 2). For kisspeptin, the data were less clear, but significant follicle size categories were in a similar size range.

**Table 2:** Generalized linear model of the number of oocytes and the number of mature oocytes retrieved by follicle diameter.

Follicle diameter (mm)	hCG	GnRH agonist (GnRHa)	Kisspeptin	hCG	GnRHa	Kisspeptin
	
	Oocytes			Mature oocytes		
	*P*-value	*P*-value	*P*-value	*P*-value	*P*-value	*P*-value
8	0.8	0.5	0.2	0.5	0.5	0.05*
9	0.2	0.7	0.1	0.1	0.8	0.2
10	0.1	0.2	0.9	0.2	0.1	0.9
11	0.3	0.02*	0.4	0.2	0.009**	0.6
12	0.02*	<0.0001***	0.03*	0.1	<0.0001***	0.04*
13	<0.0001***	<0.0001***	0.2	<0.0001***	<0.0001***	0.5
14	<0.0001***	<0.0001***	0.02*	<0.0001***	<0.0001***	0.5
15	<0.0001***	<0.0001***	0.8	<0.0001***	<0.0001***	0.7
16	<0.0001***	<0.0001***	0.9	<0.0001***	<0.0001***	0.4
17	<0.0001***	<0.0001***	0.007**	<0.0001***	<0.0001***	0.03*
18	<0.0001***	0.04*	0.1	<0.0001***	0.01*	0.7
19	0.006**	0.008**	0.9	0.1	<0.0001***	0.8
20	0.006**	0.6	0.2	0.02*	0.7	0.3
21	0.9	0.06	0.9	0.7	0.03*	0.6
22	0.1	0.6	0.7	0.4	0.3	0.9
23	0.2	0.4	0.7	0.9	0.3	0.9
24	0.1	0.9	0.7	0.4	0.9	0.6
25	0.9	0.9	0.5	0.9	0.9	0.2

P-values denote the tail area in a two-tailed Wald statistic (z value) test for the hypothesis that the associated model coefficient is 0 (and hence is unimportant when using that follicle diameter to predict number of oocytes and mature oocytes). Hence, a significantly important follicle diameter is expected to have a significantly low P-value. Follicle diameter significance levels are labeled *P < 0.05, **P < 0.01, and ***P < 0.001.

The regression coefficient (*r*^2^) for the generalized linear regression model to determine the number of oocytes retrieved for hCG was 0.49 for oocytes and 0.44 for number of mature oocytes retrieved. The regression coefficient (*r*^2^) for the generalized linear regression model to determine the number of oocytes retrieved for GnRHa was 0.56 for oocytes and 0.64 for number of mature oocytes retrieved. The regression coefficient (*r*^2^) for the generalized linear regression model to determine the number of oocytes retrieved for kisspeptin was 0.50 for oocytes and 0.39 for number of mature oocytes retrieved.

Results from random forest analysis were consistent with the generalized linear regression model suggesting that follicle sizes of 12–19 mm on the day of trigger had the greatest predictive importance for the number of oocytes and number of mature oocytes retrieved (see Table 3).

**Table 3:** Model importance factors (random forest) for follicle diameter in determining number of oocytes and number of mature oocytes.

Follicle diameter (mm)	hCG	GnRH agonist (GnRHa)	Kisspeptin	hCG	GnRHa	Kisspeptin
	
	Oocytes			Mature oocytes		
	Model importance factor	Model importance factor	Model importance factor	Model importance factor	Model importance factor	Model importance factor
8	18.9	1.3	**177.3**	25.8	0.7	**241.4**
9	42.2	0.2	**102.8**	48.0	0.3	**107.0**
10	41.4	19.8	66.1	34.7	23.3	77.5
11	38.7	54.8	**103.4**	35.9	53.2	88.5
12	**104.9**	**158.9**	88.8	**113.5**	**150.4**	84.9
13	**148.9**	**109.8**	**172.6**	**197.8**	66.1	**143.7**
14	**296.3**	**340.7**	**197.1**	**239.8**	**340.9**	**110.7**
15	**207.8**	**335.9**	58.8	**215.7**	**360.0**	77.6
16	**190.7**	**172.8**	**222.6**	**187.2**	**154.3**	**312.0**
17	**255.8**	**287.5**	**220.3**	**256.3**	**312.8**	**214.9**
18	**189.5**	**132.4**	**107.5**	**186.5**	**124.4**	77.0
19	**111.2**	**109.4**	41.2	**102.4**	**151.7**	47.6
20	71.6	46.8	51.9	92.3	36.8	42.9
21	28.2	20.0	29.5	26.3	16.6	30.9
22	30.6	7.8	19.4	22.5	7.5	32.7
23	12.0	1.9	23.5	7.1	1.0	19.7
24	11.3	0.0	22.4	8.3	0.0	18.8
25	0.0	0.0	15.4	0.0	0.0	18.7

*Model importance factors are the increases in node purity given by each follicle diameter averaged over 5,000 classification trees, each derived using bootstrap samples from the study data. Values over 100 (in bold) denote follicle diameters that are consistently important as predictors across multiple models, and hence strengthen the *P*-value results given in Table 2 which could result from a chance configuration of the study data*.

Equivalent results for the relationship between follicle sizes on day of trigger and the number of embryos and grade 1 blastocysts are presented in Table 4 (generalized linear model) and Table 5 (random forest model).

**Table 4:** Generalized linear model—number of embryos and number of high grade embryos.

Follicle diameter (mm)	hCG	GnRH agonist (GnRHa)	Kisspeptin	hCG	GnRHa	Kisspeptin
	
	Embryos			Grade 1 embryos		
	*P*-value	*P*-value	*P*-value	*P*-value	*P*-value	*P*-value
8	0.67	0.3	0.05*	0.3	0.9	0.2
9	0.2	0.8	0.005*	0.5	0.8	0.04*
10	0.1	0.02*	0.3	0.1	0.2	0.8
11	0.8	0.3	0.9	0.7	0.9	0.9
12	0.8	0.02*	0.2	0.2	0.3	0.2
13	0.1	0.0004***	0.6	0.1	0.2	0.3
14	0.001**	<0.0001***	0.6	0.6	0.002**	0.2
15	0.03*	<0.0001***	0.9	0.1	0.5	0.9
16	<0.0001***	<0.0001***	0.3	0.6	0.06	0.5
17	0.01*	<0.0001***	0.09	0.8	0.06	0.1
18	0.006**	0.01*	0.7	0.9	0.09	0.7
19	0.5	0.04*	0.7	0.2	0.01*	0.9
20	0.007**	0.3	0.3	0.5	0.8	0.2
21	0.3	0.2	0.6	0.6	0.6	0.4
22	0.4	0.7	0.3	0.5	0.4	0.6
23	0.9	0.04*	0.5	0.8	0.6	0.5
24	0.3	0.9	0.5	0.8	0.9	0.2
25	0.9	0.9	0.5	0.9	0.9	0.5

P-values denote the tail area in a two-tailed Wald statistic (z value) test for the hypothesis that the associated model coefficient is 0 (and hence is unimportant when using that follicle diameter to predict number of embryos and Grade 1 embryos). Hence, a significantly important follicle diameter is expected to have a significantly low P-value. Follicle diameter significance levels are labeled *P < 0.05, **P < 0.01, and ***P < 0.001.

**Table 5:** Model importance factors (random forest) for follicle diameter in determining number of embryos and number of high quality embryos.

Follicle diameter (mm)	hCG	GnRH agonist (GnRHa)	Kisspeptin	hCG	GnRHa	Kisspeptin
	
	Embryos			Grade 1 embryos		
	Model importance factor	Model importance factor	Model importance factor	Model importance factor	Model importance factor	Model importance factor
8	19.9	0.6	**195.0**	31.6	0.5	**238.8**
9	47.6	0.4	**144.2**	26.1	0.5	164.2
10	53.8	47.8	74.1	**161.7**	48.3	83.7
11	51.3	51.9	71.9	67.0	50.7	69.4
12	**130.3**	**166.2**	80.2	**106.2**	**160.9**	**112.6**
13	**136.5**	89.3	**160.5**	87.1	93.0	**154.4**
14	**197.9**	**354.1**	**121.4**	**244.2**	**352.5**	**204.2**
15	**167.1**	**320.0**	67.9	**132.1**	**319.0**	77.2
16	**221.2**	**180.0**	**394.5**	**191.9**	**184.8**	**235.0**
17	**235.1**	**232.1**	**177.1**	**146.7**	**233.9**	**151.2**
18	**180.3**	**172.5**	57.3	**211.4**	**176.6**	66.2
19	79.8	80.6	50.5	**111.2**	78.9	48.3
20	**151.6**	51.1	43.6	**138.3**	49.8	51.7
21	88.3	39.7	27.2	78.0	36.5	23.7
22	29.7	11.9	34.6	53.3	12.1	39.4
23	5.5	1.7	21.0	7.6	1.6	22.5
24	4.1	0.0	17.8	5.7	0.0	23.8
25	0.0	0.0	19.0	0.0	0.0	21.3

*Model importance factors are the increases in node purity given by each follicle diameter averaged over 5,000 classification trees, each derived using bootstrap samples from the study data. Values over 100 (in bold) denote follicle diameters that are consistently important as predictors across multiple models, and hence strengthen the *P*-value results given in Table 4 which could result from a chance configuration of the study data*.

### Impact of the Proportion of Follicles Within the Size Range 12–19 mm on the Number of Oocytes Retrieved

Comparing the 50% of patients with the highest proportion of their follicles within the size range 12–19 mm with the 50% with the lowest proportions in this range revealed an increase in the number of oocytes of 3.6 oocytes and 3.3 mature oocytes (*P* < 0.0001) for hCG and 3.9 oocytes and 2.5 mature oocytes (*P* < 0.01) for GnRHa triggering. Importantly, there was not a significant difference in the total number of follicles in the one group compared with the other group (20.9 follicles vs 19.3 follicles, *P* = 0.17) to account for this difference.

Repeating this analysis comparing patients with the upper tertile for proportion of their follicles within the range 12–19 mm with the lowest tertile revealed an increase in the number of oocytes of 5.4 oocytes and 4.7 mature oocytes (*P* < 0.0001) for hCG and 4.8 oocytes and 4.9 mature oocytes (*P* < 0.01) for GnRHa triggering. For GnRHa, there was no significant difference in the total number of follicles in each tertile (20.5 vs 19.1, *P* = 0.16), but in the hCG group there was an increase of 1.7 more follicles on the day of trigger between the tertiles (15.1 vs 13.4; *P* < 0.01). While this is a potential confounder, this would be insufficient to explain the magnitude of increase observed.

**Figure 1:**
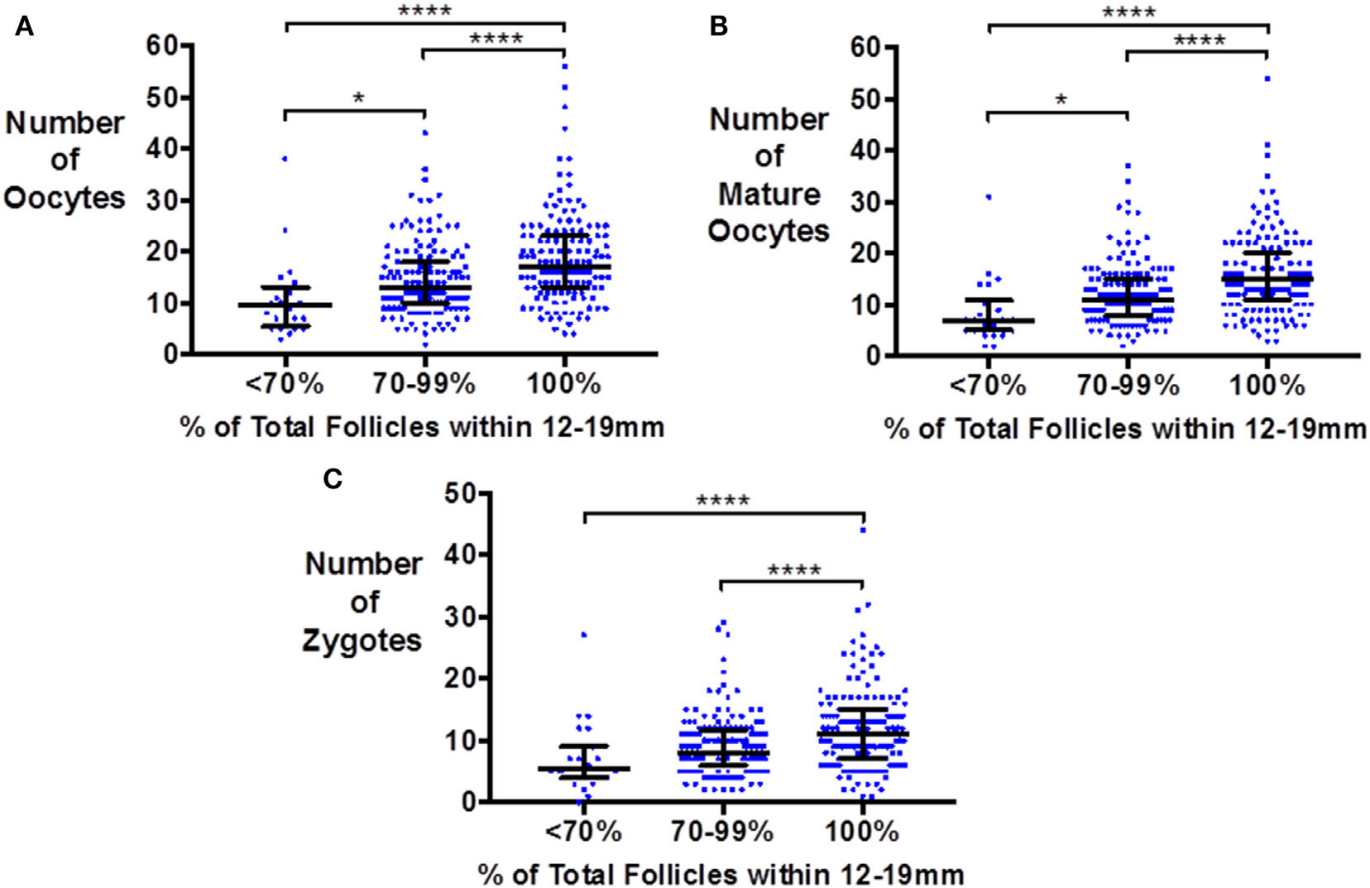
Scattergram (median and interquartile range) of the number of oocytes **(A)**, mature oocytes **(B)**, and zygotes **(C)** by the proportion of follicles on the day of trigger within the size range 12–19 mm in patients triggered with either hCG or GnRH agonist. Groups are compared by the Kruskal–Wallis test with *post hoc* Dunn’s correction for multiple comparisons (*P < 0.05 and ****P < 0.0001).

**Figure 2:**
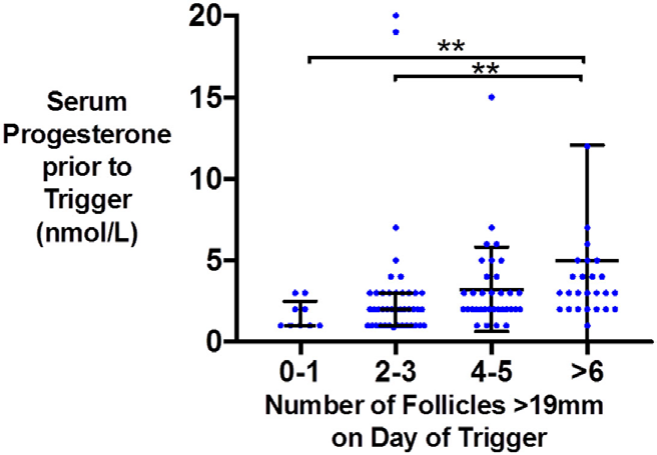
Scattergram (median and interquartile range) of serum progesterone (nmol/l) just prior to trigger administration by the number of follicles >19 mm on the day of kisspeptin trigger administration.

Similarly, the number of oocytes, mature oocytes, and zygotes, was greater by the proportion of follicles within the follicles size range 12–19 mm on day of trigger for patients triggered with either hCG or GnRHa (see Figure 1). Furthermore, delaying triggering could result in more follicles > 19 mm which in turn could increase the risk of premature rise in serum progesterone (see Figure 2).

### Comparison of Simulated Patients to Determine the Expected Importance of Follicle Size on the Number of Oocytes Retrieved Following Trigger

Using the model generated through random forest analysis of patients triggered with hCG or GnRHa, we simulated 1,000 patients having all their follicles within the optimal follicle size range (12–19 mm) and compared these to a further 1,000 simulated patients with all their follicles outside of this size range. Our model predicts that the number of oocytes retrieved from patients with 20 follicles on the day of trigger would increase from a mean of 9.8 (95% prediction limit 9.3–10.3) to 14.8 (95% prediction limit 13.3–16.3) oocytes due to the difference in follicle size profile alone (*P* < 0.001).

## Discussion

It is widely accepted that the maturity and competence of oocytes change with the size of follicles during controlled ovarian stimulation (1). Follicles that are either “too small” or “too large” are less likely to yield mature oocytes (1). To date, the size of follicles that are most likely to yield mature oocytes has predominantly been investigated on the day of oocyte retrieval (2, 18–22). Rosen et al. observed that the odds of retrieving mature oocytes from follicles of 13–15 mm on the day of oocyte retrieval were 70% less compared with follicles > 18 mm (23). Wittmaack et al. observed that follicles with a volume < 1 ml (~12.4 mm) and >7 ml (~23.7 mm) had a statistically lower oocyte yield (59%) when compared with those between 1 and 7 ml (~74–85%) (21). Dubey et al. observed that fertilization rates are increased in oocytes from larger follicles on the day of oocyte retrieval (57.9% from 10 to 14 mm follicles, 69.9% from 16 to 22 mm follicles, 73.9% from 22 to 26 mm follicles) (22). However, Ectors et al. (2) observed that follicles of 16–23 mm on the day of oocyte retrieval had higher fertilization rates (68%) than either follicles < 16 mm (56%) or those >23 mm (56%) (2). In that study, oocyte maturation rate rose from 75.3% from follicles < 16 mm, to 85.9% of follicles 16–23 mm, to 95.3% of follicle size > 23 mm (2). Overall, follicles of 16–22 mm on the day of oocyte retrieval are more likely to contain mature oocytes than smaller follicles, while larger follicles are more likely to contain post-mature oocytes (1). However, limited data exist to establish which follicle size on the day of trigger is most likely to yield a mature oocyte.

In most centers, triggering is administered once two to three ovarian follicles are at least 17–18 mm in diameter. Therefore, ultrasound folliculograms used to determine the size of follicles on the morning of trigger are usually carried out 2 days prior to those used to determine the size of follicles on the day of oocyte retrieval. As follicles grow ~1.7 mm per day, follicle sizes presented in this study would be expected to be ~3–4 mm smaller than comparable studies assessing follicular sizes on the day of oocyte retrieval (1, 24). We determine that follicles of 12–19 mm on the day of trigger administration had the greatest contribution to the number of oocytes retrieved. This is consistent with the current literature which suggests that follicles of sizes 16–22 mm on the day of oocyte retrieval (measured 2 days later) contribute the most to the number of oocytes retrieved (1).

Some studies of follicle size on the day of oocyte retrieval have suggested that there are differences in fertilization rates or oocyte competence with follicle size (22, 25). Thus, one could hypothesize that while all follicles of sizes between 12 and 19 mm contributed to the number of oocytes retrieved, perhaps only oocytes derived from larger follicles in this range (e.g., 16–19 mm) would contribute to the number of zygotes and embryos formed. However, our analyses suggested that the sizes of follicles that contributed to the formation of embryos and high quality embryos were comparable to those contributing to oocytes and mature oocytes (see Tables 4 and 5).

As this study was a non-interventional analysis and triggering was carried out once two to three ovarian follicles reached ≥17–18 mm in diameter as per routine unit protocols, there were fewer follicles at larger sizes in this study. This was consistent with the work of Dubey and colleagues, whereby 85% of oocytes were collected from follicles of 14–24 mm at the time of oocyte retrieval (22). Consequently, we investigated whether patients having a greater proportion of their follicles within the size range 12–19 mm on the day of trigger were likely to retrieve more oocytes than patients with the smallest proportion of follicles within this follicles size range. We observed that the tertile of patients with the highest proportion of oocytes within this size range on the day of trigger, retrieved ~50% more oocytes than patients with the lowest proportion of their follicles within this range. Importantly, this was not sufficiently explained by differences in the total number of follicles on the day of trigger.

As follicles increase beyond a certain size, they are more likely to yield post-mature oocytes. Furthermore, delaying triggering until follicles grow to a larger size could also result in an untimely rise in serum progesterone that could prematurely mature the endometrium, resulting in an out of phase endometrium and reduced implantation rates (26). In this study, we observe that pre-trigger serum progesterone was more likely to be elevated if there were a greater number of larger follicles (≥19 mm) on the day of trigger (see Figure 2). One could speculate that in addition to the size of follicles, the duration at which larger follicles are present before trigger administration and whether effective GnRH antagonism has been achieved could also contribute to the degree of premature progesterone elevation. Similarly, Kolibianakis observed that delaying the trigger by 48 h resulted in 1.3 fewer follicles of 11–14 mm and 3.1 more follicles of ≥17 mm with an associated rise in progesterone of 0.4 ng/ml and detrimental effects on pregnancy potential (27). Kyrou et al. compared administering the trigger once three follicles were ≥16 mm in diameter (early), or 24 h later (late), and found that delaying triggering increased the number of mature oocytes retrieved (early 6.1, late 9.2, *P* = 0.009) with an associated rise in serum progesterone levels by 0.3 ng/ml (28). Mochtar and colleagues randomized women to receive trigger once the lead follicle was either 18 or 22 mm, and observed that those with a lead follicle of 22 mm had a greater number of follicles of 20–22 mm on day of trigger (3.95 vs 0.02) and an increase in two oocytes retrieved (29). Conversely, Tan and colleagues randomized patients to trigger either once the lead follicle was 18 mm, or 1 day later, or 2 days later and observed no differences in the number of oocytes retrieved (30). Similarly, Tremellen and Lane found that patients with “ideal” timing of the hCG trigger (defined as ≥2 follicles of ≥17 mm, with the majority of follicles ≥14 mm) had similar outcomes to patients triggered either a day earlier or later (31), whereas Vandekerckhove et al. observed that a 24 h delay in trigger administration of patients with ≥3 follicles of ≥18 mm (and 30–50% of follicles ≥10 mm were also ≥1 8 mm) increased the number of mature oocytes retrieved by 2.4, but only in patients with a serum progesterone ≤1 ng/ml (32). A meta-analysis by Chen et al. including 7 RCTs and 1,295 IVF cycles compared administration of hCG as soon as ≥3 follicles were ≥17 mm in size (“early”) vs administration of hCG either 24 or 48 h later (“late”) (33). While fertilization rates were higher in the 48 h later group (*P* < 0.0001), this result was predominantly attributable to the results of one study, and overall there was no significant benefit from later triggering (33).

Lessons on the size of follicle from which mature oocytes can be retrieved can also be learned from studies of *in vitro* maturation (IVM) (34). Follicles as small as 4 mm have been found to contain mature oocytes, and mature oocytes from follicles ≤10 mm following hCG priming resulted in similar outcomes compared with those retrieved from larger follicles (35). However, the rate of *in vivo* matured oocytes positively correlated with follicle size (dominant follicle ≤10 mm 6.9%, 10–14 mm 10.6%, >14 mm 15.1%) (36). Finally, Triwitayakorn et al. observed that oocyte recovery rate increased from 57% of follicles < 10 mm to 80% of follicles 10–14 mm and further to 86% of follicles > 14 mm on the day of oocyte retrieval (37).

Kisspeptin has only recently been investigated as a trigger of oocyte maturation since 2014; consequently, data from the kisspeptin trials may have incorporated doses which were suboptimal for oocyte maturation. Thus, while similar results were observed for kisspeptin as for other triggers, it is interesting to note that some smaller follicles could also contribute to the number of oocytes retrieved for kisspeptin more so than for other triggers (see Tables 2 and 3). Although the contribution was small, several studies have suggested that kisspeptin may have additional direct ovarian effects *via* ovarian kisspeptin receptors, beyond its predominant mode of action *via* endogenous GnRH release from the hypothalamus (38–41). Commensurate with this, Castellano observed that kisspeptin expression increased in a cyclical manner during the menstrual cycle of a rodent model, predominantly localized to the theca layer of growing follicles and the corpora lutea (38). Ovarian kisspeptin expression was undetectable in immature oocytes, but increased at ovulation (38). Kisspeptin has been reported to enhance IVM of sheep oocytes (39) and also of porcine oocytes, as well as blastocyst formation rate and blastocyst hatching (40). However, while it is possible to speculate that kisspeptin could enhance oocyte maturation in combination with gonadotropin exposure, it is unlikely that *in vivo* administration can lead to oocyte maturation in the absence of a gonadotropin-response (9).

Although the present study included patients with a large number of oocytes retrieved, we do not advocate the use of an hCG trigger in the high risk patient with multiple follicles, especially if fresh embryo transfer is intended to be carried out, and we definitely promote the use of GnRHa trigger for oocyte donation cycles. Limitations of the study include that is a non-interventional retrospective analysis. Further randomized studies are required to determine whether triggering of oocyte maturation once most follicles are within the size range 12–19 mm can lead to improved oocyte yields compared with traditional determination of day of triggering. Furthermore, as data from hCG and GnRHa trigger were obtained from cycles without fresh embryo transfer, it was not possible to assess the reproductive potential of oocytes obtained from follicles of different sizes. The current method of determining the day of trigger administration once two to three lead follicles are 17–18 mm in size should lead to a similar day of trigger as most follicles will still be within the size range 12–19 mm. However, determining the day of trigger based on the proportion of follicles within the size range 12–19 mm could be of particular value to patients with a wider spread of follicles behind the lead follicle. In addition, we recommend that these analyses be re-conducted in data sets obtained from different centers with the possibility of different stimulation protocols or study populations to confirm the results from this study.

In summary, we conclude that follicles of 12–19 mm on the day of trigger are most likely to yield mature oocytes on the day of oocyte retrieval. Thus, we recommend the reporting of mature oocyte yields using a denominator of follicle size of 12–19 mm on the day of trigger for studies investigating trigger efficacy. Future interventional studies should investigate whether using the proportion of follicles within 12–19 mm to determine the day of trigger administration could improve the number of mature oocytes retrieved.

## Ethics Statement

Data included in this manuscript were obtained from studies carried out in accordance with the recommendations of the local ethical boards listed below. All subjects gave written informed consent in accordance with the Declaration of Helsinki and Good Clinical Practice. Data from GnRHa triggered IVF cycles were obtained from a single-center randomized controlled trial conducted at My Duc Hospital, Ho Chi Minh City, Vietnam (11). The Institutional Review Board (IRB) reference number was NCKH/CGRH_01_2014, and ClinicalTrials.gov registration was NCT02208986. For the hCG case-series, the IRB reference number was NCKH/CGRH_09_2017, ethical approval reference number: 10/17/DD-BVMD, and ClinicalTrials.gov Identifier: NCT03174691. For the kisspeptin trial, ethical approval was granted by the Hammersmith and Queen Charlotte’s Research Ethics Committee, London, UK (reference: 10/H0707/2), undertaken at the IVF Unit at Hammersmith Hospital under a license from the UK Human Fertilization and Embryology Authority (9, 11, 12) and registered on the National Institutes of Health Clinical Trials database (NCT01667406).

## Author Contributions

All authors provided contributions to study conception and design, acquisition of data or analysis and interpretation of data, drafting the article or revising it critically for important intellectual content, and final approval of the version to be published. Here are the most important contributions of each author: AA, LV, RS, TK, GT, PH, and WD designed the study. Data were collected by AA, LV, VH, TK, SC, LJ, AC, and TH. Analysis was carried out by AA and TK. PH and WD take final responsibility for this article.

## Conflict of Interest Statement

Trial of GnRHa was sponsored by Merck Sharp & Dohme (grant number IIS 52023). PH declares unrestricted research grants from MSD, Merck and Ferring as well as honoraria for lectures from MSD, Merck and Finox. There are no other competing interests to declare.
